# MicroRNA Expression Analysis: Clinical Advantage of Propranolol Reveals Key MicroRNAs in Myocardial Infarction

**DOI:** 10.1371/journal.pone.0014736

**Published:** 2011-02-28

**Authors:** Wenliang Zhu, Lei Yang, Hongli Shan, Yong Zhang, Rui Zhou, Zhe Su, Zhimin Du

**Affiliations:** 1 Institute of Clinical Pharmacology, The Second Affiliated Hospital of Harbin Medical University, Harbin, China; 2 The First Affiliated Hospital of Harbin Medical University, Harbin, China; 3 Department of Pharmacology, Harbin Medical University, Harbin, China; Harvard School of Public Health, United States of America

## Abstract

**Background:**

As playing important roles in gene regulation, microRNAs (miRNAs) are believed as indispensable involvers in the pathogenesis of myocardial infarction (MI) that causes significant morbidity and mortality. Working on a hypothesis that modulation of only some key members in the miRNA superfamily could benefit ischemic heart, we proposed a microarray based network biology approach to identify them with the recognized clinical effect of propranolol as a prompt.

**Methods:**

A long-term MI model of rat was established in this study. The microarray technology was applied to determine the global miRNA expression change intervened by propranolol. Multiple network analyses were sequentially applied to evaluate the regulatory capacity, efficiency and emphasis of the miRNAs which dysexpression in MI were significantly reversed by propranolol.

**Results:**

Microarray data analysis indicated that long-term propranolol administration caused 18 of the 31 dysregulated miRNAs in MI undergoing reversed expression, implying that intentional modulation of miRNA expression might show favorable effects for ischemic heart. Our network analysis identified that, among these miRNAs, the prime players in MI were miR-1, miR-29b and miR-98. Further finding revealed that miR-1 focused on regulation of myocyte growth, yet miR-29b and miR-98 stressed on fibrosis and inflammation, respectively.

**Conclusion:**

Our study illustrates how a combination of microarray technology and functional protein network analysis can be used to identify disease-related key miRNAs.

## Introduction

Increasing lines of evidences suggest that microRNAs (miRNAs) are involved in modulating cardiac processes and in the progression of heart disease [1]. Because of the large number of miRNAs, microarray technology has been extensively applied in revealing global miRNA expression changes in human models of heart disease associated with cardiac hypertrophy, heart failure, and myocardial infarction [2]. In the present study, we proposed a microarray based network biology approach to identify the key miRNAs most likely related to myocardial infarction (MI) by integrating miRNA expression and protein-protein interaction (PPI) network data.

MI is characterized by a loss of excitability associated with ionic, functional, and metabolic abnormalities. Many clinical trials have verified that antiarrhythmic drugs such as sodium-channel, potassium-channel or calcium-channel blockers cannot prevent the morbidity associated with MI induced malignant arrhythmias [3], [4]. In contrast, β-adrenoceptor blockers have proven successful in preventing sudden cardiac death in patients with MI [5]-[7]; however, the essential mechanism underlying the beneficial effects of these agents remains unclear. As a vital node in posttranscriptional regulation of many biological functions, miRNAs are considered as important regulators of human physiological and pathological conditions [8]. Consequently, it is entirely possible and perhaps likely that the alterations of miRNA expression may be involved in the beneficial therapeutic effects of agents such as propranolol, a β-adrenoceptor blocker. In order to determine the miRNAs most highly related to MI, we employed miRNA chips to evaluate the global miRNA expression of a MI rat model produced by the occlusion of the left anterior descending coronary artery. Propranolol was chronically administered to induce reversal of MI and global expression change of miRNAs expressed in rat heart was determined. Multiple network analyses were then employed to quantitatively access the regulatory capacity, efficiency and emphasis of the miRNAs which dysexpression in MI were significantly reversed by propranolol.

In a recent review, Pan et al. discussed the possibility of taking miRNAs as potential therapeutic targets for treating cardiac diseases, especially ischemic heart disease. Such an opinion was proposed that successful reversal of pathological processes can only be obtainable by modulating some key regulators [9]. Through miRNA microarray and protein interaction network data integration, our approach presented here could not only pick out key miRNAs involved in MI but also bring novel insight in the pathological mechanisms of MI from the miRNA aspect. Intriguingly, as a vital node in posttranscriptional regulation, miRNAs participate in many cellular pathways in both physiological and pathological conditions [10]. Therefore, undoubtedly, the method presented here is also applicable to other human diseases.

## Results

### Propranolol affected miRNA expression profile of MI

**Figure 1:**
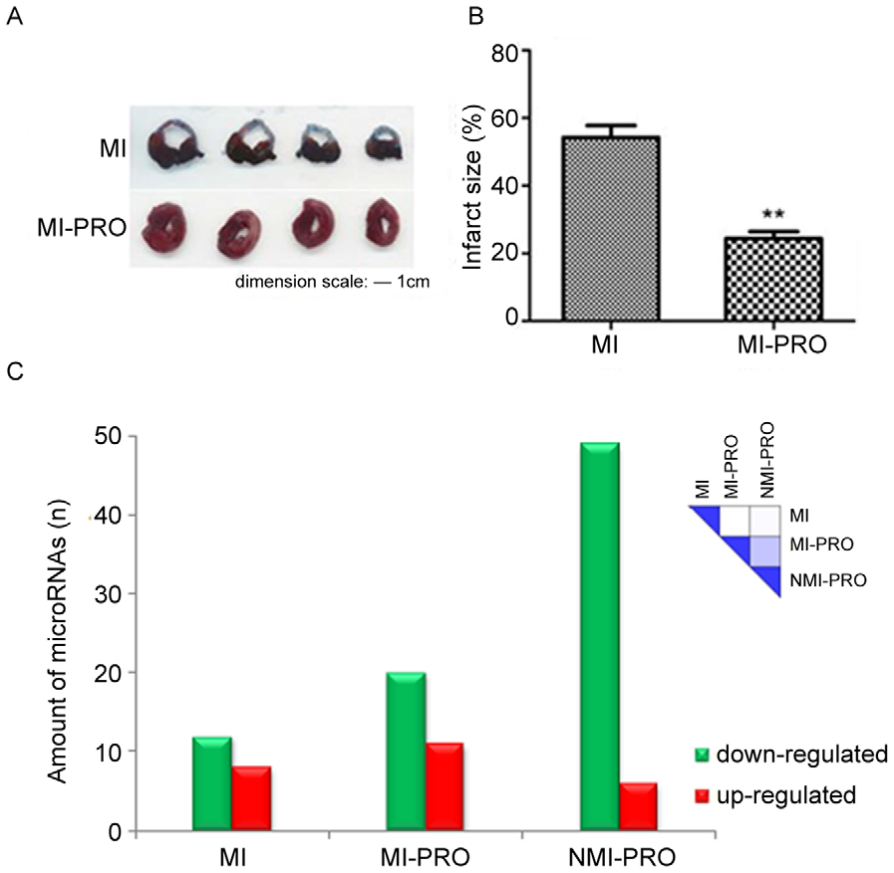
Effects of propranolol on the infarct size and miRNAs expression. A. Effects of propranolol on the infarct size in rats with myocardial infarction. Left panel: the white portion of the coronal sections of the left ventricular wall represents the infarct area. Right panel: the size of infarct area was expressed as the percentage of a standard coronal section. **p<0.01 vs. MI (two-way paired t-test), n = 5. B. Count of miRNAs with expression levels varied by at least ±0.5 from the levels in Control. The right upper heatmap shows the miRNA expression similarity between groups. MI, myocardial infarction; MI-PRO, myocardial infarction-propranolol; NMI-PRO, non-myocardial infarction-propranolol. C. Quantification of miR-1 expression. Relative level of miR-1 normalized to control; ***p<0.0001 vs. control; ##*p*<0.01 vs. MI (two-way paired t-test), n = 8.

The beneficial effect of propranolol on MI was investigated using four randomized groups of experimental rats: control, myocardial infarction (MI), MI with propranolol treatment (MI-PRO) and non-MI with propranolol treatment (NMI-PRO). Oral administration of propranolol at a daily dose of 10 mg/kg significantly reduced the infarct size of the left ventricle in rats with MI (Figure 1A). Among the 102 miRNAs which can be detected in all miRNA arrays, almost half of them underwent obvious downregulation in NMI-PRO group while only 12 miRNAs did so in MI group (Figure 1B). The propensity of miRNA targeting positive regulatory motifs [11] implied the prodigious miRNA expression change was consistent with the favorable effects of propranolol on MI (Figure 1B). The expression similarity of 0.39 (MI-PRO versus NMI-PRO) suggested the benefits of propranolol to MI were associated with the rectified miRNA expression profile (Figure 1B, right upper). Among the 31 dysregulated miRNAs in MI (Table S1), 18 PRmiRs (propranolol-reversed miRNAs, PRmiRs) were found in MI rats administered with propranolol (Fold change>1.5, MI-PRO versus MI).

### Static topological analysis identified miRNAs with enhanced regulation

**Figure 2:**
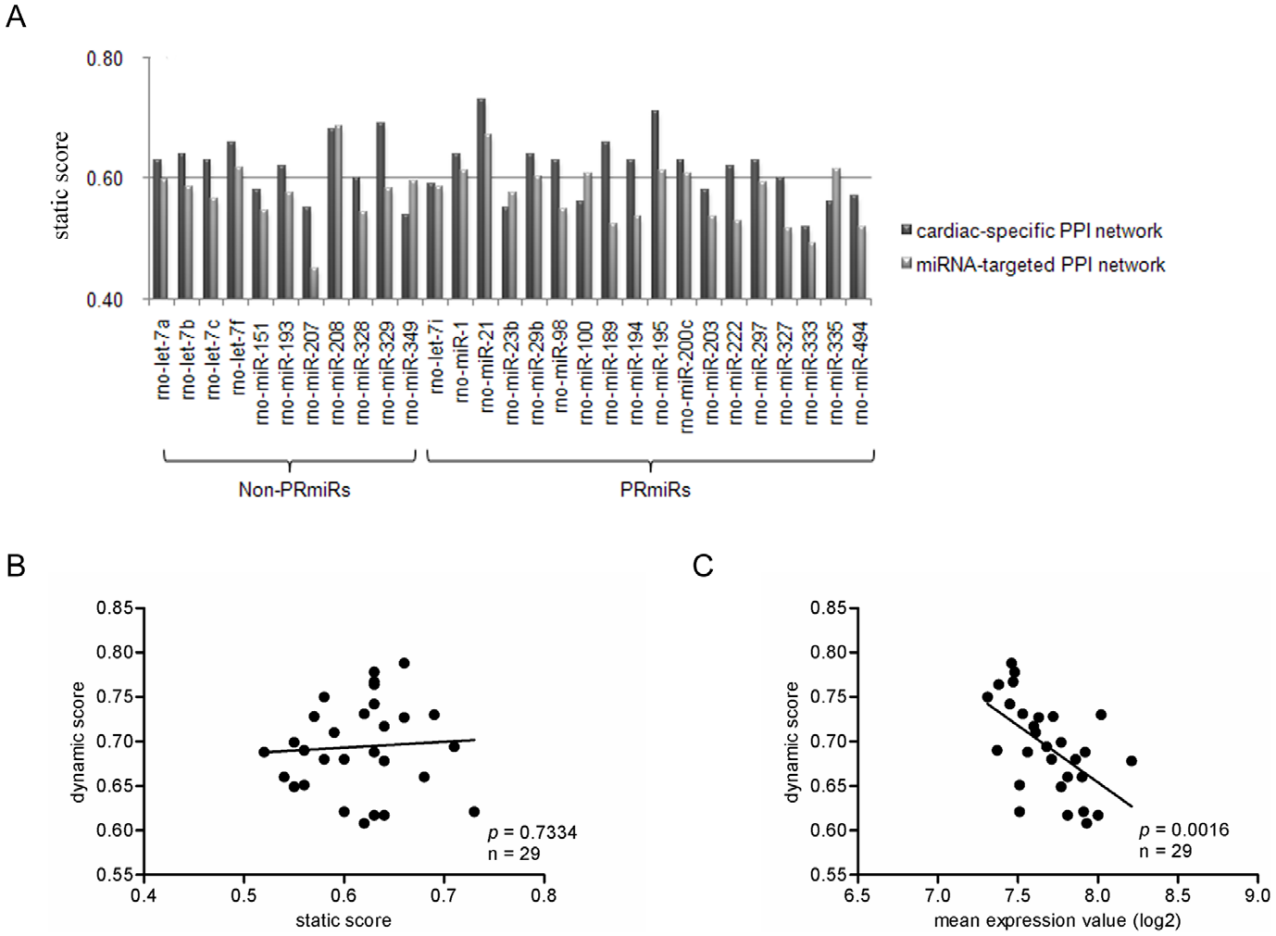
Results of static and dynamic network analyses. A. Static scores of the 29 dysregulated miRNAs in myocardial infarction. B. Correlation between static score and dynamic score. C. Correlation between average target gene abundance (log2) and dynamic score. non-PRmiR, dysregulated miRNA in myocardial infarction which expression could not be reversed by propranolol; PRmiR, propranolol-reversed miRNAs.

To perform static topological analysis, a cardiac-specific PPI network was established in advance by importing heart expressed genes of MI Wistar rats (accession number: GDS808) [12] into STRING [13]. The final network was comprised of 2846 nodes and 22163 interactions, when determined at the relatively high confidence level of 0.7. After the validated and predicted target genes [14], [15] associated with the MI-dysregulated miRNAs listed in Table S1 were imported into the network, the Cytoscape [16] plug-in NetworkAnalyzer [17] was applied for calculating static score (See MATERIALS AND METHODS). There were 10 PRmiRs and 7 non-PRmiRs assigned with static scores of more than 0.6 suggesting enhanced regulation by them (Figure 2A). To further confirm the result of static analysis upon the cardiac-specific network, a larger PPI network was also established by importing the target genes of all the chip detected miRNAs into STRING. As a result, we obtained the miRNA-targeted PPI network, which was comprised of 4940 nodes and 88776 interactions at the same confidence level of 0.7. Similarly, we calculated the static scores of the MI-dysregulated miRNAs (Figure 2A). Notably, only 4 PRmiRs showed strengthened regulation in both of the PPI networks. They were miR-1, miR-21, miR-195 and miR-200c. And 2 non-PRmiRs let-7f and miR-208 did so.

To investigate whether strengthened control made miRNAs more efficient to overcome the robustness of the PPI network itself, we performed a dynamic perturbation analysis to virtually simulate the effect of altered miRNA expression on global free protein concentration in the cardiac-specific PPI network. The gene expression data (accession number: GDS808) was imported into the network as substitute nodes as attributed by ProteinAbundance [18]. As dynamic score was introduced here (See MATERIALS AND METHODS), the Cytoscape plug-in PerturbationAnalyzer quantified the regulatory efficiency of the MI-dysregulated miRNAs against the network robustness. Interestingly, we found that dynamic scores were significantly associated with the mean target gene abundances (log2, *p* = 0.0016), instead of static scores (Figure 2B and C). This result implied that excessive target gene abundance could dilute the regulatory performance of a miRNA, even though the important roles of its interacted genes in biological regulation might be found [19]. Among the 18 PRmiRs, miR-98 showed the most efficient gene regulation (dynamic score: 0.778, Figure 2C). As a static score of 0.63 indicated its enhanced regulation on the cardiac-specific PPI network, abnormal expression of miR-98 in MI might strongly imbalance its controlled biological modules.

### ClusterOne analysis discovered the regulatory emphasis of miRNAs

**Figure 3:**
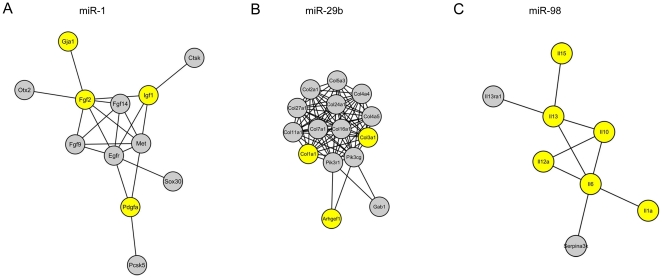
Results of ClusterOne analysis. A-C. Largest functional modules regulated by miR-1, miR-29b and miR-98, respectively. The yellow nodes represent proteins encoded by genes that are associated with ischemia.

With an opinion that a miRNA with too scattering gene regulation might not likely induce significant phenotype, the ClusterOne Cytoscape plug-in [20] was used to disclose the enriched functional module that was comprised of proteins encoded by target genes of the 10 PRmiRs which enhanced regulation was found on the cardiac-specific PPI network (Figure 3). With the Gene Prospector tool in the HuGE Navigator database [21] applied, we established the association between the modules and disease phenotype ischemia. The modules regulated by miR-1, miR-29b and miR-98 contained more proteins encoded by ischemia related genes (Figure 3). The DAVID functional annotation clustering tool [22] then identified the significantly over-represented biological process each module was involved in (Figure 3). The result demonstrated that miR-1 stressed upon regulation of myocyte growth, yet miR-29b and miR-98 put their regulatory emphases upon fibrosis and inflammation, respectively.

## Discussion

In recent years, miRNAs have been identified as key regulators of protein expression and have been implicated in many physiologically relevant cellular pathways [8]. Altered miRNA expression has been implicated in the cardiovascular system and changes in miRNAs have been correlated with cardiac diseases [23]. In the present study, an innovative integrative analysis of protein-protein interaction and miRNA expression data was undertaken to search for the miRNAs that might be key regulators of MI, a common cause of heart failure. In particular, we employed a long-term propranolol-administered rat model of MI with a notion that the favorable effects of propranolol, a β-adrenoceptor blocker, could provide us some useful clues. MiRNA microarray analysis showed that the alteration of miRNAs expression was consistent with the favorable effects of propranolol (Figure 1A and B). Further finding revealed that propranolol successfully reversed the altered expression of two-thirds of the dysregulated miRNAs in MI (Table S1).

To quantitatively describe gene regulation of miRNAs, we introduced two parameters static and dynamic scores. As the cardiac-specific and miRNA-targeted PPI networks were established by retrieving protein interactions of high confidence scores derived by benchmarking the performance of the predictions against KEGG [24], reference set of associations from STRING [13], static score inherently scaled the regulatory capacity of each dysregulated miRNAs (Figure 2A). Two topological parameters degree and neighborhood connectivity were integrated into the calculation of static score. By counting the number of neighbors connected to a given node, the parameter degree reflects its status in cellular functions [25]. Similarly, high neighborhood connectivity of a node means the likelihood that it is connected with a highly connected protein and involved in important cellular functions [26]. If static score of 0.6 reflected moderate regulation by a miRNA, five PRmiRs and two non-PRmiRs performed enhanced gene regulation upon both of the PPI networks (Figure 2A). To access the regulatory efficiency of each dysregulated miRNAs, a dynamic perturbation analysis was designed by applying the PerturbationAnalyzer Cytoscape plug-in [18], a convenient tool for calculating the perturbation effect in a PPI network by comparing equilibrium states. To perform such a simulation, the concentrations of proteins in the PPI network need to be known. Unfortunately, the genome-wide protein abundances in the rat are not yet available [18]. Instead, we used gene expression data found in the GEO database [12] as an approximation of protein abundance for PertubationAnalyzer calculations. This rationale for this approach is based, in part, on the previous findng that in yeast a direct relationship exists between mRNA levels and protein levels [27]. The result of dynamic perturbation simulation suggested that efficient gene regulation executed by a miRNA was inversely correlated with its mean target gene abundance and not associated with its regulatory capacity in essential (Figure 2B and C). Our finding was consistent with the previous work of Arvey et al [19].

To investigate the regulatory emphasis of individual PRmiRs with enhanced regulation upon the cardiac-specific PPI network, the ClusterOne analysis was performed to discover the functional PPI modules they strongly controlled (Figure 3). The DAVID functional annotation clustering tool [22] and the gene evidence retrieved from the HuGE Navigator database [21] jointly identified three PRmiRs which were more closely related to MI (Figure 3). They were miR-1 emphasizing on cell growth regulation, miR-29b stressing on fibrosis and miR-98 focusing on inflammation. Approximately 60% of the target genes of them were assigned comparatively small p-values or experimentally validated (See MATERIALS AND METHODS). This made our conclusion credible (Table S2).

There are increasing lines of evidences to suggest that miR-1 might be a vital regulator of MI [28], [29]. Lu et al. confirmed that the administration of propranolol could reverse the expression of miR-1 in ischemic rat heart and inhibition of miR-1 could provide ischemic cardioprotection [30]. Taken together, these studies provide functional links between miR-1 and MI, which is consistent with its higher static score in our network analysis. It was recently demonstrated that miR-29b was dramatically down-regulated in the region of the fibrotic scar after MI and collagen expression in the heart was modestly increased in response to miR-29b inhibition [31]. In contrast, we found an elevation in miR-29b. Although the important role of miR-98 in inflammatory response of cholangiocyte has been verified [32], the experimental evidence of its involvement in inflammation during myocardial ischemia still lacks. In conclusion, while the PPI network presented here may include some false positive interactions and false prediction of miRNA gene targets, it is hoped that such a systemic analysis can help confirm and identify the key miRNAs that was related to MI.

## Materials and Methods

### Drugs and animals

Propranolol (Sigma-Aldrich, St Louis, MO, USA) was firstly dissolved in 20% dimethyl sulfoxide (DMSO), and then diluted in PBS to the final concentration before the experiment. The final concentration of DMSO was 0.02% and shown no effects on cardiac functions (data not shown). Other reagents were purchased from Sigma (St Louis, MO, USA). All procedures involving the use of animals in this study complied with the regulations and protocols of the ethic committees of Harbin Medical University and the Guide for the Care and Use of Laboratory Animals of the US National Institutes of Health (NIH Publication No. 85–23, revised 1996). Male Wistar rats (250–300 g) were conditioned for one week at room temperature, with 55±5% humidity and a 12 h cycle of light/dark. They were allowed free access to food and tap water. The rats were randomly divided into 4 groups: control, myocardial infarction (MI), myocardial infarction-propranolol (MI-PRO), and non-myocardial infarction-propranolol (NMI-PRO). All the rats were anesthetized with pentobarbital (40 mg/kg, i.v.), and then rat MI model was established by occluding left anterior descending coronary artery under sterile conditions. A daily oral dose of 10 mg/kg of propranolol was administered to MI-PRO and NMI-PRO rats for 2 months before the experiment. After the rats were sacrificed, the infarct zone of the left ventricle was rapidly isolated and used for the miRNA microarray experiment. The open chest procedures were also experienced by control and NMI-PRO animals, but the coronary artery occlusion was not applied to them.

### Myocardial infarct size measurement

Size of the infarct area of the left ventricle in rats was measured as described previously [33].

### MiRNA microarray

The microarray assay for miRNAs profiling was conducted by the China Shanghai Kangcheng Technology Co, Ltd. In total, left ventricular samples of eight rat hearts from each group were pooled and in the end four miRNA chips were prepared. The microarray data is MIAME compliant (accession number: GSE18129). Between groups (MI, MI-PRO and NMI-PRO), expression similarities of miRNAs with expression levels varied by at least ±0.5 from the levels in control (dysregulated miRNA, dmiR) were calculated as followed:
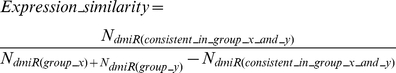



### Static and dynamic network analyses

To perform static and dynamic network analyses upon miRNAs, two PPI networks were established in this study. The cardiac-specific PPI network was built by retrieving relatively high confident protein interactions (confidence score: 0.7) from STRING and gene expression data for MI Wistar rat hearts from GEO (accession number: GDS808). To build the miRNA-targeted PPI network, the target genes of miRNAs detected by the miRNA chip were retrieved from Microcosm and their protein products were combined for online search for PPIs in STRING. Again, the confidence score of 0.7 was chosen. Notably, for STRING did not permit long list submission, we disassembled the entire list. The network depth of 2 was chosen so that every direct neighboring node could be involved in the downloaded sub-network. The final merged network was then established by applying Cytoscape 6.2.3.

To evaluate the regulatory capacity of individual miRNAs, the Cytoscape plug-in NetworkAnalyzer was applied to calculate two topological parameters degree and neighborhood connectivity for every node in the two networks. After importing the information of the validated [14] and predicted [15] target genes of a miRNA into the networks, the normalized mean degree and neighborhood connectivity values of the nodes representing proteins encoded by its target genes were calculated with network mean value as background. Finally, the miRNA obtained an integrated parameter static score as the sum of the normalized mean degree and neighborhood connectivity values was subtracted by 3.3. Higher static score means stronger regulation exerted by a miRNA. In Microcosm, *p*-value is computed to identify significant correlation between miRNA and target genes by considering the genomic miRanda score distribution. If a *p*-value is small, it means that the corresponding prediction result is likely correct [34]. For each analyzed miRNA, the percentage of validated and more correctly predicted genes (*p*-value<0.01) in the networks was calculated.

To evaluate the regulatory efficiency of a miRNA against the network robustness, the gene expression data of MI Wistar rat hearts (accession number: GDS808) was imported in the network as the substitute node attribute of ProteinAbundance. The Cytospcape plug-in PerturbationAnalyzer was used to access the global effect of altered miRNA expression upon network homeostasis as proteins encoded by its targeted genes were selected as perturbation sources in the manual perturbation mode. After perturbation, the ratio of the property Perturbed subgroup size at 2.0 and 1.2 subgroup thresholds was calculated as dynamic score. A low dynamic score means that network robustness can more easily resist the impact of altered miRNA expression.

### ClusterOne analysis

To disclose the regulatory emphasis of a given miRNA, the Cytoscape plug-in ClusterOne was applied to discover the significantly overlapping regions in the PPI network which was only comprised of proteins encoded by its target genes. The PPIs were retrieved from STRING as confidence score of 0.7 was chosen. As the default settings of ClusterOne were unchanged, the largest module within each network was selected for further biological process analysis. The DAVID functional annotation clustering tool was responsible for investigating the significantly over-represented biological process proteins in the module might participate in. The functional annotation ‘GOTERM_BP_ALL’ was only selected. Meanwhile, the Gene Prospector tool in the HuGE Navigator database was applied to explore the experimentally validated association between their corresponding genes and the disease-phenotype ischemia.

## Supporting Information

Table S1 The 31 dysregulated miRNAs in myocardial infarction.
(0.05 MB DOC)

Table S2 The percentage of experimentally validated and high confidently predicted target genes (p-value < 0.01).
(0.05 MB DOC)
