# Supplementary material for: MicroRNA Expression Analysis: Clinical Advantage of Propranolol Reveals Key MicroRNAs in Myocardial Infarction
Source: PLoS One. 2011 Feb 28;6(2):e14736. doi: 10.1371/journal.pone.0014736 (PMC3046111; doi:10.1371/journal.pone.0014736)
Supplement: Table S1 — The 31 dysregulated miRNAs in myocardial infarction. (0.05 MB DOC) [file pone.0014736.s001.doc]

Supplementary table S1. The 31 dysregulated miRNAs in myocardial infarction

| Category | miRNA | Expression ratio (MI/control) | Expression ratio (MI-PRO/control) | Expression ratio (MI-PRO/MI) |
| --- | --- | --- | --- | --- |
| non-PRmiR | rno-let-7a | 0.52 | 0.51 | 0.98 |
|  | rno-let-7b | 0.59 | 0.72 | 1.21 |
|  | rno-let-7c | 0.50 | 0.57 | 1.13 |
|  | rno-let-7f | 0.53 | 0.67 | 1.26 |
|  | rno-miR-122a | 0.64 | 0.79 | 1.24 |
|  | rno-miR-151 | 0.52 | 0.46 | 0.89 |
|  | rno-miR-193 | 0.43 | 0.43 | 1.00 |
|  | rno-miR-207 | 1.53 | 1.76 | 1.15 |
|  | rno-miR-208 | 0.65 | 0.55 | 0.84 |
|  | rno-miR-328 | 3.19 | 2.37 | 0.74 |
|  | rno-miR-329 | 1.57 | 1.54 | 0.98 |
|  | rno-miR-349 | 1.59 | 1.15 | 0.72 |
|  | rno-miR-422b | 0.53 | 0.66 | 1.25 |
| PRmiR | rno-let-7i | 0.24 | 0.87 | 3.61 |
|  | rno-miR-1 | 1.68 | 0.38 | 0.23 |
|  | rno-miR-21 | 2.95 | 0.36 | 0.12 |
|  | rno-miR-23b | 1.60 | 0.64 | 0.40 |
|  | rno-miR-29b | 1.70 | 0.28 | 0.17 |
|  | rno-miR-98 | 0.55 | 1.15 | 2.07 |
|  | rno-miR-100 | 0.23 | 0.52 | 2.20 |
|  | rno-miR-189 | 0.44 | 0.78 | 1.76 |
|  | rno-miR-194 | 0.45 | 0.72 | 1.59 |
|  | rno-miR-195 | 0.59 | 1.28 | 2.16 |
|  | rno-miR-200c | 1.66 | 0.87 | 0.53 |
|  | rno-miR-203 | 0.37 | 0.74 | 2.00 |
|  | rno-miR-222 | 0.30 | 0.97 | 3.28 |
|  | rno-miR-297 | 0.64 | 1.44 | 2.25 |
|  | rno-miR-327 | 1.93 | 1.27 | 0.66 |
|  | rno-miR-333 | 0.51 | 0.81 | 1.58 |
|  | rno-miR-335 | 2.07 | 0.58 | 0.28 |
|  | rno-miR-494 | 1.72 | 0.77 | 0.45 |

The 31 dysregulated miRNAs in myocardial infarction. MI, myocardial infarction; MI-PRO, myocardial infarction-propranolol; non-PRmiR, dysregulated miRNA in myocardial infarction which expression could not be reversed by propranolol; PRmiR, propranolol-reversed miRNAs.
