# Supplementary material for: MicroRNA Expression Analysis: Clinical Advantage of Propranolol Reveals Key MicroRNAs in Myocardial Infarction
Source: PLoS One. 2011 Feb 28;6(2):e14736. doi: 10.1371/journal.pone.0014736 (PMC3046111; doi:10.1371/journal.pone.0014736)
Supplement: Table S2 — The percentage of experimentally validated and high confidently predicted target genes (p-value < 0.01). (0.05 MB DOC) [file pone.0014736.s002.doc]

Supplementary table S2. The percentage of experimentally validated and high confidently predicted target genes (*p*-value < 0.01)

| Category | miRNA | Percentage (cardiac-specific PPI network) | Percentage (miRNA-targeted PPI network) |
| --- | --- | --- | --- |
| non-PRmiR | rno-let-7a | 75.6% | 81.2% |
|  | rno-let-7b | 76.6% | 76.9% |
|  | rno-let-7c | 76.6% | 79.1% |
|  | rno-let-7f | 72.9% | 73.7% |
|  | rno-miR-122a | - | - |
|  | rno-miR-151 | 46.7% | 45.1% |
|  | rno-miR-193 | 50.4% | 51.8% |
|  | rno-miR-207 | 48.7% | 49.4% |
|  | rno-miR-208 | 53.8% | 58.6% |
|  | rno-miR-328 | 54.1% | 53.6% |
|  | rno-miR-329 | 47.9% | 46.3% |
|  | rno-miR-349 | 56.6% | 47.9% |
|  | rno-miR-422b | - | - |
| PRmiR | rno-let-7i | 76.2% | 76.8% |
|  | rno-miR-1 | 62.2% | 61.9% |
|  | rno-miR-21 | 60.2% | 60.9% |
|  | rno-miR-23b | 55.7% | 56.9% |
|  | rno-miR-29b | 62.3% | 65.2% |
|  | rno-miR-98 | 66.7% | 58.5% |
|  | rno-miR-100 | 44.9% | 47.7% |
|  | rno-miR-189 | 51.4% | 44.9% |
|  | rno-miR-194 | 57.3% | 58.2% |
|  | rno-miR-195 | 55.2% | 54.0% |
|  | rno-miR-200c | 73.6% | 75.0% |
|  | rno-miR-203 | 55.1% | 59.1% |
|  | rno-miR-222 | 55.9% | 54.0% |
|  | rno-miR-297 | 41.7% | 47.0% |
|  | rno-miR-327 | 46.5% | 43.3% |
|  | rno-miR-333 | 50.0% | 43.4% |
|  | rno-miR-335 | 47.0% | 45.2% |
|  | rno-miR-494 | 40.4% | 39.3% |

The percentage of experimentally validated and high confidently predicted target genes (*p*-value < 0.01). non-PRmiR, dysregulated miRNA in myocardial infarction which expression could not be reversed by propranolol; PRmiR, propranolol-reversed miRNAs. -, Percentage could not be calculated for Microcosm did not predict target genes of the miRNA.
